# Detection of circulating tumor DNA without a tumor-informed search using next-generation sequencing is a prognostic biomarker in pancreatic ductal adenocarcinoma

**DOI:** 10.1016/j.neo.2021.06.005

**Published:** 2021-07-21

**Authors:** Kajsa E. Affolter, Sabine Hellwig, David A. Nix, Mary P. Bronner, Alun Thomas, Carrie L. Fuertes, Cindy L. Hamil, Ignacio Garrido-Laguna, Courtney L. Scaife, Sean J. Mulvihill, Hunter R. Underhill

**Affiliations:** a Department of Pathology, University of Utah, Salt Lake City, Utah; b ARUP Laboratories, Salt Lake City, Utah; c Huntsman Cancer Institute, University of Utah, Salt Lake City, Utah; d Department of Family and Preventative Medicine, Divisions of Genetic Epidemiology and Public Health, University of Utah, Salt Lake City, Utah; e Department of Surgery, University of Utah, Salt Lake City, Utah; f Department of Pediatrics, Division of Medical Genetics, University of Utah, Salt Lake City, Utah; g Department of Radiology, University of Utah, Salt Lake City, Utah

**Keywords:** Pancreatic ductal adenocarcinoma, Next-generation sequencing, Cell-free DNA, Circulating tumor DNA, Biomarker

## Abstract

The confounding effects of next-generation sequencing (NGS) noise on detection of low frequency circulating tumor DNA (ctDNA) without *a priori* knowledge of solid tumor mutations has limited the applications of circulating cell-free DNA (ccfDNA) in clinical oncology. Here, we use a 118 gene panel and leverage ccfDNA technical replicates to eliminate NGS-associated errors while also enhancing detection of ctDNA from pancreatic ductal adenocarcinomas (PDACs). Pre-operative ccfDNA and tumor DNA were acquired from 14 patients with PDAC (78.6% stage II-III). Post-operative ccfDNA was also collected from 11 of the patients within 100 days of surgery. ctDNA detection was restricted to variants corresponding to pathogenic mutations in PDAC present in both replicates. PDAC-associated pathogenic mutations were detected in pre-operative ccfDNA in four genes (*KRAS, TP53, SMAD4, ALK*) from five patients. Of the nine ctDNA variants detected (variant allele frequency: 0.08%-1.59%), five had a corresponding mutation in tumor DNA. Pre-operative detection of ctDNA was associated with shorter survival (312 vs. 826 days; $\chi^{2}$=5.4, *P* = 0.021). Guiding ctDNA detection in pre-operative ccfDNA based on mutations present in tumor DNA yielded a similar survival analysis. Detection of ctDNA in the post-operative ccfDNA with or without tumor-informed guidance was not associated with outcomes. Therefore, the detection of PDAC-derived ctDNA during a broad and untargeted survey of ccfDNA with NGS may be a valuable, non-invasive, prognostic biomarker to integrate into the clinical assessment and management of patients prior to surgery.

## Introduction

As cells undergo apoptosis, the DNA released into the bloodstream without a protective membrane has been termed circulating cell-free DNA (ccfDNA). The discovery of ccfDNA originating from a solid tumor (i.e., circulating tumor DNA, ctDNA) was first achieved in 1994 using PCR-based methods to target a specific mutagenic locus – the *KRAS* G12 codon [1]. Targeted PCR-based methods remained the principal tool for ctDNA detection until 2012 when the first applications of next-generation sequencing (NGS) in ccfDNA were described [2,3]. Compared to PCR-based methods, NGS affords the opportunity to broadly survey ccfDNA for ctDNA without *a priori* knowledge of specific tumor mutations. However, integration of NGS into ccfDNA diagnostics has generally found greater success by focusing on a narrow set of well-defined mutagenic loci or using NGS to catalogue somatic tumor DNA mutations to guide the subsequent PCR-based searches for ctDNA [4,5]. Although these approaches have substantially advanced ccfDNA as a diagnostic tool, the full potential of NGS as the primary method to detect ctDNA remains limited.

The challenges with ctDNA detection are illustrated by prior efforts to identify activating *KRAS* mutations in ccfDNA associated with pancreatic ductal adenocarcinomas (PDACs). *KRAS* mutations in the G12, G13, and Q61 codons occur in >90% of PDACs [6,7]. Thus, the absence of ctDNA detection relates to either the tumor not actively shedding ctDNA or the amount of ctDNA being below the limit of detection by NGS rather than a biologic discordance between tumor DNA and ctDNA as described in other solid tumors [4]. Regardless, detection of PDAC-derived activating *KRAS* mutations in ctDNA has varied widely with reported NGS sensitivities ranging between 0% and 100% [5,[8], [9], [10], [11], [12]]. Notably, detection variability persists even when identical methodologies are applied to different patient cohorts with similar extents of disease [8,9]. The reason for the disparity between different studies is challenging to elucidate in part because the vocabulary that has emerged in association with NGS can confound understanding of different approaches. Therefore, to establish terminology for the study presented herein, we briefly describe the two principal components that govern ctDNA detection by NGS irrespective of study design: ctDNA signal and NGS-associated noise.

The signal from ctDNA is governed by theoretical and experimental constraints. As a reference point, each 10 ng of ccfDNA contains ~2,800 human genomic equivalents (using an average weight of 650 Da per base pair and a genomic length of 3.3 × 10^9^ base pairs), which represents the theoretical maximum obtainable unique observation read-depth for each 10 ng of ccfDNA input used during NGS library preparation. Adapter ligation efficiency for ccfDNA has been reported at 55% to 80% [13], [14], [15], which reduces the number of available unique ccfDNA molecules and lowers the experimental maximum read-depth to ~1,500-2,300X assuming no additional losses. In practical terms, a ctDNA variant with an allele frequency of 0.1% would on average be represented by 2 unique reads for each 10 ng of ccfDNA input. Increasing the amount of ccfDNA to boost the ctDNA signal is limited by the relatively low yield of ccfDNA from the finite amount of patient plasma. Although cancer patients tend to have more ccfDNA per mL of plasma, the quantity of ccfDNA in early-stage and non-metastatic cancer patients overlaps with the quantity in healthy controls [16]. In prior NGS studies seeking to detect ctDNA associated with pancreatic cancer, the ccfDNA input has ranged between <5 ng to 30 ng [5,[8], [9], [10],^12^], which translates to a maximum experimental read-depth of less than 1,000X to 7,000X.

NGS-associated noise largely arises from PCR errors, sequencing, and alignment artifacts. Integration of unique molecular identifiers (UMIs) into adapter technology has enabled substantial noise reduction via *in silico* analysis – aligned PCR amplicons with the same UMI are combined to determine a single consensus sequence [17], [18], [19]. The number of unique consensus sequences at a given position is the collapsed consensus read-depth and theoretically corresponds to the quantity of unique ccfDNA molecules used as library input. To enhance UMI error correction, additional strategies based on position-specific error modeling have been proposed [13]. However, a central contributor to NGS-associated noise is early and random PCR errors that persist regardless of adapter technology [14]. Thus, error modeling alone may not be able to fully account for these stochastic PCR artifacts, which has largely constrained variant detection to tumor-informed searches [20,21]. Recently, we described the use of technical replicates during ccfDNA library generation in combination with UMIs and removal of systematic error as a robust method to mitigate NGS-associated noise [14]. However, the impact of using technical replicates for variant detection (i.e., sensitivity) in ccfDNA to identify both known and unknown tumor-associated mutations has not been previously described.

Here, we use technical replicates, two libraries prepared and sequenced independently from each extracted ccfDNA sample, to simultaneously suppress NGS-related noise and amplify the ctDNA signal associated with PDAC. Technical replicates are initially integrated with UMIs and position-specific error modeling to probe for variants in the *KRAS* G12, G13, and Q61 codons to demonstrate the challenges with ctDNA detection at even the most well-studied tumorigenic loci. Subsequently, we show that NGS-associated errors can be effectively eliminated, thereby enabling the unbiased search for ctDNA across an entire 118 gene panel that includes genes commonly associated with PDACs: *KRAS, SMAD4, CDKN2A*, and *TP53* [22]. Finally, we show that detection of PDAC-derived ctDNA in pre-operatively acquired ccfDNA with or without *a priori* knowledge of somatic tumor mutations predicts patient survival.

## Materials and Methods

### Participants, sample collection, pathology, and DNA isolation

All procedures were approved by the University of Utah Internal Review Board prior to study initiation (protocol #89989). Healthy adult controls with no previous history of cancer, inflammatory disease, or other chronic medical condition, and patients presenting to the Huntsman Cancer Institute at the University of Utah with a pancreatic mass for a pre-operative evaluation were recruited for study enrollment. Pregnant women were excluded from the study. All study participants provided written informed consent.

Whole blood was collected in BCT tubes (Streck, La Vista, NE). Buffy coat and plasma were separated via centrifugation at 1,900 g x 20 minutes at room temperature and aspirated to new tubes. Plasma underwent a second centrifugation at 12,100 g x 10 minutes at 4° C to remove any cellular debris. Buffy coat and plasma were stored at -80° C within 24 hours of sample acquisition. The QIAamp DNA Blood Mini Kit (Qiagen, Germantown, MD) was used to isolate white blood cell (WBC) DNA from the buffy coat. The QIAamp Circulating Nucleic Acid Kit (Qiagen) was used to isolate cell-free DNA from plasma. Tumor tissue was formalin-fixed and paraffin-embedded (FFPE). All 14 tumors were PDACs and staged using the 8th edition American Joint Committee on Cancer criteria. Using an H&E slide, the study surgical pathologists subspecializing in pancreatic pathology identified the densest regions of adenocarcinoma, which were subsequently macrodissected to enrich for tumor DNA. Tumor DNA was extracted using the QIAamp DNA FFPE Tissue Kit (Qiagen).

### Library preparation and sequencing

All subsequent methods associated with tumor DNA and ccfDNA were performed in separate replicate procedures. 100 ng of WBC DNA and 100 ng of tumor DNA were used as library input. WBC DNA and tumor DNA were sheared using a focused ultrasonicator (S220, Covaris, Woburn, MA) with a targeted size of 175 bp. Two mL plasma equivalents of ccfDNA from pancreatic tumor patients was targeted for library input. Because of a low initial plasma volume and insufficient quantity of ccfDNA, less was used for the second replicate in one patient (Table S1). In some of the post-operative samples, the yield was high and an upper limit of 150 ng for library input was applied (Table S1). To provide a sufficiently complex sample from the four healthy controls, 20 ng of ccfDNA was used as library input. The Kapa HyperPrep Kit (Roche, Indianapolis, IN) and Kapa Library Amplification Kit (Roche) were used for end-repair, A-tailing, adapter ligation, and PCR amplification. Adapters consisted of a single index and single 8-mer UMI (Integrated DNA Technologies [IDT], Coralville, IA). Libraries underwent panel capture enrichment using a custom-designed capture probe set (118 genes, 123 kb; IDT) [14] followed by paired-end sequencing (125 × 2 bp) on a HiSeq 2500 (Illumina, San Diego, CA). The number of paired fastq reads for ccfDNA are detailed in Table S1.

### Bioinformatics

Procedures associated with alignment to hg19/GrCh37 and consensus calling are identical to that previously described [14]. Briefly, PCR amplicons with the same unclipped start position were grouped based on 100% 8-mer UMI similarity. A consensus sequence was determined by examining each base position in the sequencing stack – those with >0.66 base concordance were assigned the predominant base and the maximum quality score, otherwise, an N base was assigned with zero quality. Each of the tools used for alignment and consensus calling are publicly available in USeq tools (https://github.com/HuntsmanCancerInstitute/USeq) and integrated in several containerized snakemake workflows (https://github.com/HuntsmanCancerInstitute/Workflows/tree/master/Hg38RunnerWorkflows/DnaConsensusAlignQC, CtSomaticCaller, and Annotator).

Aligned consensus sequences with a family size ≥2 were selected from bam files using the USeq tool SamAlignmentExtractor. All subsequent steps and analyses used the extracted bam file containing aligned consensus sequences with family size ≥2. Samtools mpileup (version 1.9) was used to measure read-depth at each position. For variant detection, a workflow was generated that used paired WBC DNA (normal) and ccfDNA or tumor DNA associated with each participant. For each bam, vcf files were first generated with bcftools mpileup and norm. The bcftool output vcf files were passed to the USeq tool SimpleSomaticCaller to identify likely somatic variants using a Fisher's Exact test. Filters were set to the corresponding maximum or minimum values to allow all single nucleotide variants (SNVs) that varied from the germline WBC DNA to pass regardless of counts and allele frequency. Aligned consensus sequences with family size ≥2 from this data set were generated and used to model the position-specific error using the USeq tool VCFBkz. For each variant identified by SimpleSomaticCaller, a Z-score based on allele frequency was generated, where the Z-score value represented the number of standard deviations from the mean variant allele frequency in a pool of normals – ccfDNA data from seven healthy controls previously sequenced using the identical adapters, gene panel, and sequencer [14]. Only exon variants were included in the analysis. To mitigate potential artifacts associated with clonal hematopoiesis of indeterminate potential (CHIP) [23], including occurrence of CHIP with <2% allele frequency because of the read-depths achieved, a variant was excluded if present in both WBC DNA and tumor/ccfDNA unless the variant allele frequency in the tumor/ccfDNA was five-fold higher than WBC DNA. Lastly, the COSMIC database [24] was used to adjudicate potential variants in ccfDNA. Only variants that matched to ‘pathogenic’ or ‘likely-pathogenic’ and ‘pancreas’ were retained.

### Statistics

The Chi-square test was used to compare homogeneity of proportions. Student's t-test was used for between group comparisons using either the paired or unpaired version as appropriate for the sampling design. Pearson's correlation was used to analyze the association between groups. Survival analysis was performed using a log rank test (Mantel-Cox) and Kaplan-Meier curves were plotted. Results were considered statistically significant for *P* < 0.05. All statistical analyses were performed with IBM SPSS Statistics (Version 26).

## Results

### Study participants, surgical results, and ccfDNA samples

Between March 2016 and November 2018, whole blood was obtained from 14 patients (age: 68.6 ± 10.3 yrs; 28.6% female) during a pre-operative visit for PDAC. Histopathology and FFPE tumor DNA were obtained from surgical procedures intended for complete tumor resection. Complete surgical resection with tumor-free resection margin(s) (R0) was achieved in five patients and incomplete resections with tumor present microscopically at resection margin(s) (R1) or metastatic disease was present in nine patients. The demographics and pertinent treatment information for each patient are provided in Table 1. For 11 patients, whole blood was also obtained within 100 days after surgery (mean: 28.8 ± 28.3 days; range: 2 – 97 days). Overall, median clinical follow-up after initial diagnosis was 529 days. In the 11 deceased patients, follow-up ranged from 224 to 1,195 days (median: 426 days). In the three living patients, follow-up was 579, 1,090, and 1,041 days. Between October and November 2018, whole blood was collected from four healthy adult controls (age: 47.5 ± 13.9 yrs; 50% female) with no previous history of cancer, inflammatory disease, or other chronic medical condition.

**Table 1:** Patient demographics and number of days for each event after the initial diagnosis of pancreatic ductal adenocarcinoma.

Patient	Age, Sex	TNM (Stage Group)	Neo-Adjuvant Therapy	Pre-Op ccfDNA (days)	Surgery (days)	Surgical Procedure, Resection Status	Post-Op ccfDNA (days) [POD]	Status	Survival (days)
P1	53, F	ypT2N2 (III)	Gemcitabine/Nab-Paclitaxel^a^	114	222	W, R1	251 [29]	Deceased	1,195
P2	77, M	pT2N2 (III)	None	39	39	W, R1	42 [3]	Deceased	241
P3	77, M	pT3N2 (III)	None	61	61	W, R0	104 [43]	Deceased	224
P4	73, M	pT1cN1 (IIB)	None	18	18	W, R0	20 [2]	Living	1,041
P5	75, F	pT1cN2 (III)	None	41	41	W, R1	75 [34]	Deceased	292
P6	81, M	ypT3N1 (IIB)	Gemcitabine	153	153	DPS, R1	155 [2]	Deceased	418
P7	62, M	pT3N2 (III)	None	55	55	W, R0	71 [16]	Deceased	681
P8	68, M	ypT2N2 (III)	mFOLFIRNOX	197	197	W, R0	205 [8]	Deceased	826
P9	72, M	pT3N1 (IIB)	None	8	8	DPS, R0	c	Deceased	479
P10	70, M	ypT2N1 (IIB)	Gemcitabine/Nab-Paclitaxel + SBRT^b^	300	300	W, R1	c	Living	1,090
P11	57, M	pT2N2 (III)	None	53	53	W, R1	c	Living	579
P12	70, F	ypTxNxM1 (IV)	Gemcitabine/Nab-Paclitaxel	169	169	MDP	266 [97]	Deceased	312
P13	45, F	pTxNxM1 (IV)	None	0	0	MDP	49 [49]	Deceased	626
P14	69, M	ypT2N2 (III)	mFOLFIRNOX	166	166	W, R1	200 [34]	Deceased	426

DPS = distal pancreatectomy and splenectomy; MDP = metastatic disease present; POD = post-op day; R1 = margin positive for microscopic tumor; R0 = margin negative for microscopic and macroscopic tumor; SA = surgery aborted; W = Whipple procedure

a ccfDNA sample obtained during neoadjuvant therapy

b Unable to complete full course of Gemcitabine/Nab-Paclitaxel and referred for SBRT

c Sample not obtained

For each study participant, the amount of plasma used to extract ccfDNA, the ccfDNA library input amount, and corresponding plasma mL equivalents are detailed in Table S1 (Fig. S1). The concentration of ccfDNA in plasma ranged from 3.8-5.8 ng/mL plasma in controls, 3.5-30.2 ng/mL plasma in patients pre-operatively, and 3.2-67.7 ng/mL plasma in patients post-operatively.

### Prevalence of NGS error associated with activating *KRAS* G12, G13, and Q61 mutations in ccfDNA

First, we sought to characterize the presence and frequency distribution of variants present in pre-operative and control ccfDNA at each of the nine positions (hg19/GrCh37) associated with the *KRAS* amino acid coding positions G12 (chr12:25,398,283-25,398,285), G13 (chr12:25,398,280-25,398,282), and Q61 (chr12:25,380,275-25,380,277). Read-depth, which subsequently references the aligned consensus read-depth, is shown in Fig. S2 for the nine positions. Previously published sequencing data from a set of seven healthy controls that followed the same methodology [14] was used to calculate a position-specific Z-score based on the allele frequency of error. To reduce false positives associated with noise caused by PCR errors and sequencing artifacts, data from the replicates were merged to identify variants present in both (i.e., shared). The mean read-depth after combining the replicates was 7,536 ± 1,825X (range: 4,853-11,426X; Fig. S2). For each shared variant, replicate data were combined to recalculate allele frequency and the geometric mean of the Z-scores from each replicate determined the shared Z-score.

**Fig. 1:**
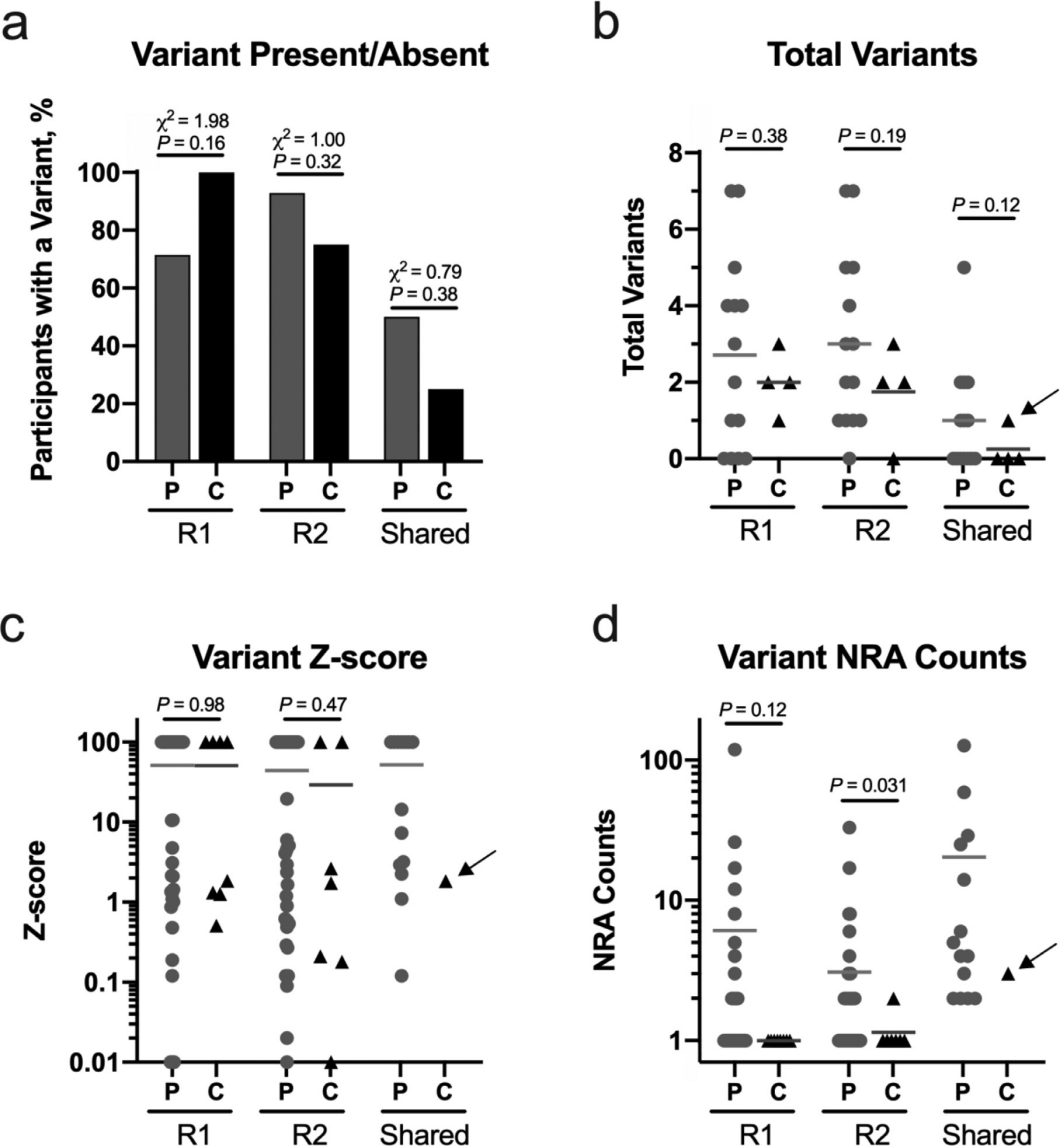
Error in *KRAS* at amino acid coding positions G12, G13, and Q61 confounds separation of cases and controls. In (A), the presence of at least one *KRAS* variant did not discriminate the pre-operative ccfDNA of 14 patients with PDAC (P; cases) from the ccfDNA of four healthy controls (C). No difference was observed between cases and controls using the total number of variants (B) and the Z-score (ie, position-specific error modeling; C) associated with each variant. In (C), note that some Z-score values are low (< 1) indicating that the allele frequency associated with that variant was similar to the noise in the pool of normal controls. Although the difference was not consistently significantly different between cases and controls in both replicates, there was evidence that the number of nonreference allele (NRA) counts associated with a variant may help distinguish signal from noise (D). The solid black arrow in B, C, and d identifies the p.G12D variant shared in both replicates from a control patient. R1 = replicate 1; R2 = replicate 2; solid bars represent the mean value.

Applying UMI technology alone to suppress error yielded prevalent noise in control ccfDNA at the nine *KRAS* positions associated with activating pathogenic mutations in PDAC. The presence and/or absence of a *KRAS* nonreference allele (i.e., potential variant or mutation) in ccfDNA did not discriminate between pancreatic tumor patients (i.e., cases) and controls in the individual replicates (Fig. 1A). Merging the replicate data to identify shared variants reduced error, but the inability to separate cases from controls persisted (Fig. 1A). We also found that the total number of variants detected, Z-score values, and nonreference allele counts associated with each variant did not separate cases from controls either (Fig. 1B-D). Collectively, these observations highlight the confounding nature of NGS-associated artifacts. Although position-specific error modeling (ie, Z-score) identifies and removes systematic patterned error, the occurrence of a single nonreference allele by chance at a position without error in the pool of normals generates a high Z-score value, which limits discerning signal from noise using Z-score alone. Similarly, technical replicates substantially reduced error, but the single shared nonreference allele from the healthy controls had a pathogenic p.G12D mutation – the most common mutation associated with PDAC [25]. Thus, we determined that additional selection criteria were necessary to comprehensively suppress error. Notably, we found compelling evidence (Fig. 1D) that introducing a threshold based on nonreference allele counts may discriminate signal from noise, particularly when applied to determination of shared variants.

### Integrated *in silico* error suppression with technical replicates

To establish a nonreference allele count threshold applicable across the entire 118 gene panel, we examined error at all exon positions in the seven reference control samples [14] used in determining position-specific error (ie, Z-score). Variants with nonreference allele counts ≤3 accounted for 95% of the false positives for the given experimental conditions (Fig. S3). Although each increment in nonreference allele count further reduced error, application of larger thresholds was not implemented to mitigate loss of sensitivity because the replicates were being combined to identify shared variants for noise reduction.

**Fig. 2:**
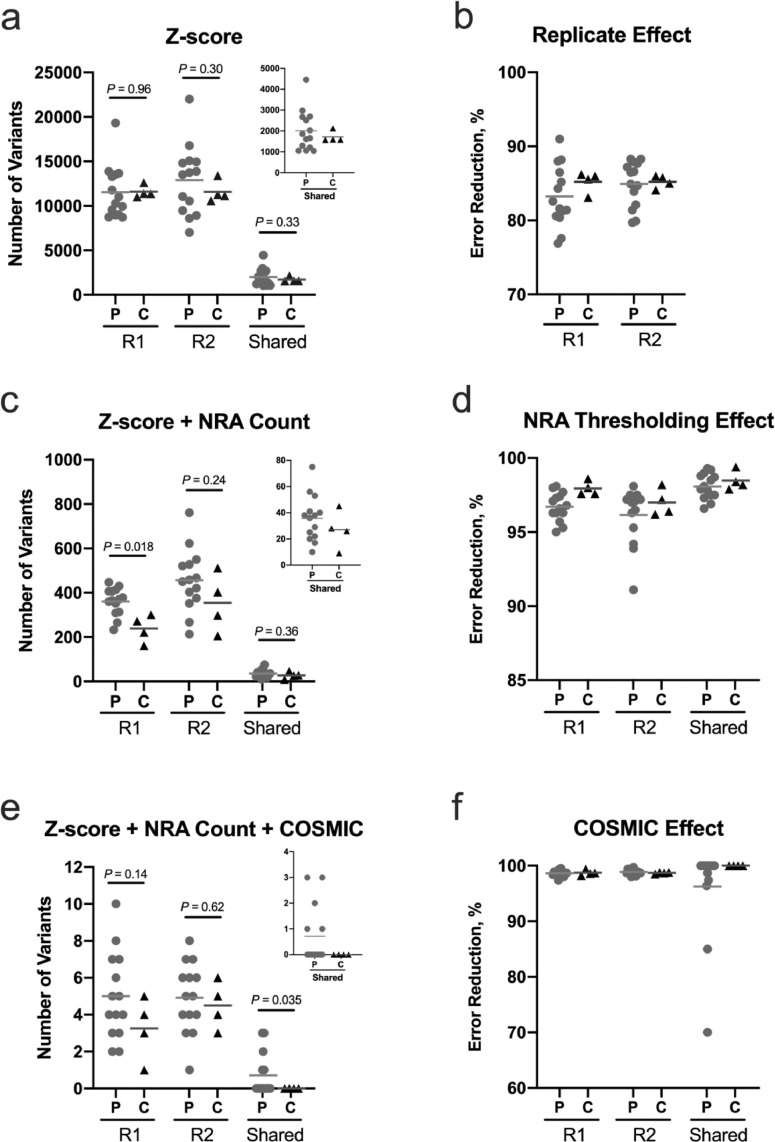
Integration of technical replicates into a multi-step *in silico* error correction strategy eliminates NGS artifacts. Data is shown for both replicates (R1, R2) for the 14 PDAC patients (P, gray circles) and four healthy controls (C, black triangles). Potential variants common to both replicates are identified as “Shared.” All data is from collapsed consensus reads using UMIs to reduce error. In (A), the baseline number of variants using a Z-score threshold of 2.576 (99^th^ percentile) to remove error is shown. Identifying variants present in both replicates reduced error in the controls by >83% (B). In (C), the number of potential variants is further reduced by applying a nonreference allele (NRA) count threshold – each identified variant had >3 NRA counts in both replicates. In the controls, this additional criterion further reduced error by >95% (D). Finally, only pathogenic variants in the COSMIC database associated with the pancreas were retained (E). Although errors persisted in individual replicates, 100% of the noise was suppressed by combining replicates (F). In A, C, and E, the inset is a magnification of the ‘Shared’ result. Note, the application of each noise elimination strategy also reduced potential variants in the PDAC patient group which may have removed true positives. R1 = replicate 1; R2 = replicate 2; solid bars represent the mean value.

The mean read-depth for the ~101,400 exonic positions associated with the 118 genes is shown in Figure S2 (Table S1). The baseline number of variants after noise reduction using UMIs and also applying a minimum Z-score value of 2.576 (99th percentile) as a threshold in the pre-operative ccfDNA from 14 PDAC patients and the ccfDNA from four healthy controls is shown in Fig. 2A. As previously described [14], combining replicates to identify shared variants reduced error >83% in the controls with a similar reduction in patients (Fig. 2B). Removal of potential variants with nonreference allele counts ≤3 further reduced the number of variants by >95% in controls (Fig. 2C). Requiring shared variants to have >3 nonreference alleles in each replicate further reduced the total number of shared variants by >98% in the controls (Fig. 2D). However, the remaining total variant count did not consistently distinguish cases from controls in the replicates or after combining replicates. Thus, the COSMIC database was used to constrain variant identification in ccfDNA to pathogenic mutations associated with the pancreas (Fig. 2E). Nine pathogenic variants were present in pre-operative ccfDNA from both replicates in five of the 14 PDAC patients (35.7%), while none were present in the controls (Fig. 2E-F). A description of each pathogenic variant associated with PDAC in the COSMIC database from the cases and controls for each replicate and the shared variants are detailed in Tables S2 and S3 and Table 2, respectively.

**Table 2:** Pancreatic ductal adenocarcinoma-associated pathogenic mutations in pre- and post-op ccfDNA

Patient	Gene	cDNA	AA	Pre-Op				Post-Op			
				NRA Count	Read-Depth	VAF, %	Z-score	NRA Count	Read-Depth	VAF	Z-score
P2	*KRAS*	c.181C>A	p.Q61K+	25	7,896	0.32	7.3	MND			
	*KRAS*	c.38G>A	p.G13D	127	7,999	1.59	100.0	MND			
P3	*KRAS*	c.35G>A	p.G12D	29	11,423	0.25	14.4	31	8,914	0.35	18.2
	*SMAD4*	c.1082G>A	p.R361H	26	12,303	0.21	6.0	16	9,927	0.16	4.4
	*TP53*	c.586C>T	p.R196X	41	11,576	0.35	33.8	28	9,681	0.29	27.2
P7	*KRAS*	c.38G>A	p.G13D	59	5,698	1.04	100.0	MND			
P12	*ALK*	c.3257C>T	p.S1086L	9	11,762	0.08	6.3	MND			
P14	*KRAS*	c.34G>C	p.G12R	14	8,746	0.16	100.0	10	9,863	0.10	100.0
	*TP53*	c.844C>T	p.R282W	19	11,036	0.17	4.5	40	11,909	0.34	9.6

AA = amino acid; MND = mutation not detected; NRA = nonreference allele; VAF = variant allele frequency

p.Q61K+ (P2) – the ‘+’ indicates there was a co-occurring c.180T>A mutation

Next, as a possible surrogate of the completeness of surgical resection, we sought to identify the presence of pathogenic variants associated with PDAC in ccfDNA acquired post-operatively using the same integrated replicate analysis to suppress NGS-associated noise. Of the nine pathogenic variants detected pre-operatively in five patients, five variants from two patients persisted post-operatively (Table 2). There was not a clear clinical explanation for disappearance or persistence of ctDNA in the post-operative samples, including the consideration of the standard gross and histologic evaluations of resection margin status. However, we observed that in the two patients with persistent detection of ctDNA the post-operative samples were collected >30 days after surgery and the concentration of ccfDNA increased <15%. In contrast, the concentration of ccfDNA in plasma increased by >180% in the three patients with loss of the ctDNA signal. Post-operative samples from two of these three patients were acquired ≤16 days after surgery suggesting an abundance of normal ccfDNA released in association with post-operative inflammation and surgery-associated tissue injury may have confounded ctDNA detection. This conjecture is supported by a reduction in the concentration of ccfDNA in plasma within the first 60 days of the post-operative period for most patients (Fig. S4). Although the third patient's sample (P12) was acquired at post-op day 97, the patient was undergoing adjuvant chemotherapy which may have suppressed ctDNA detection through release of ccfDNA from healthy cells. Overall, no new pathogenic mutations associated with PDAC were identified in the ccfDNA post-operatively. Thus, the role of postoperative ccfDNA samples, particularly in the proximal post-operative period (i.e., <30 days), remains unclear for gauging residual disease.

### ctDNA concordance with solid tumor DNA

**Fig. 3:**
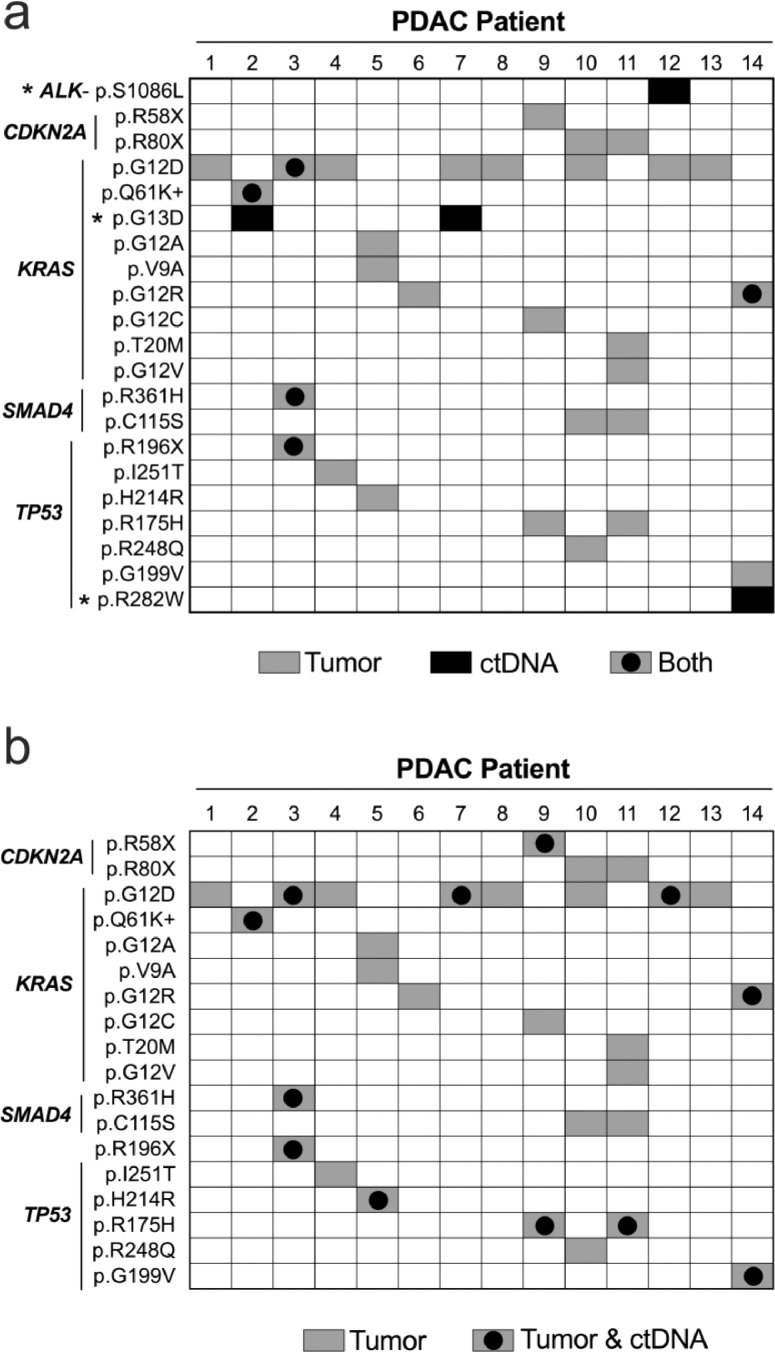
Concordance between tumor tissue DNA and pre-operative ctDNA. In (A), the ctDNA variants identified using an unbiased search via integration of technical replicates into *in silico* error suppression methods were compared to mutations present in the tumor tissue DNA. Three additional mutations were present in ctDNA (asterisks) from four patients that were absent in solid tumor DNA. In (B), using the mutations present in tumor DNA to guide the search for ctDNA improved sensitivity by allowing the replicate data to be combined to increase read-depth along with a reduction in the thresholding criterion because patient-specific variants were sought. PDAC = pancreatic ductal adenocarcinoma

Somatic mutations present in tumor DNA were catalogued for all 14 PDAC patients. A mutation in tumor DNA was identified by presence in both tumor DNA replicates and a VAF ≥1.0% after combining replicates. The mean read-depth was 3,725 ± 976X (range: 2,079-5,480X). Of the 118 genes on the panel, somatic mutations were only identified in *KRAS, SMAD4, TP53*, and *CDKN2A* (Table S4). A single G12, G13, or Q61 activating *KRAS* mutation in tumor DNA was identified in all 14 patients (100%) with a mean VAF of 18.7% ± 18.2% (range: 3.2% - 60.2%). Although additional *KRAS* mutations were identified in two patients, none had more than one activating *KRAS* mutation in tumor DNA. Five of the nine (55.6%) ctDNA variants identified using the integrated technical replicate analysis were also present in the sampled tumor tissue DNA (Fig. 3A). Four of the ctDNA variants were not detected in tumor DNA, reflecting potential limitations of using a focal tissue sample to evaluate tumor heterogeneity. Notably, the *KRAS* p.G13D activating mutation was present in ctDNA with a VAF >1% in two patients, but was absent in the corresponding tumor DNA (Table 2 and Table S4).

Next, we used mutations detected in tumor tissue DNA to guide the search for ctDNA. Because patient-specific known tumor variants were sought in corresponding ccfDNA, the replicate ccfDNA data for each patient were combined to maximize read-depth without requiring the variant be present in both replicates. Output from bcftools mpileup was used for nonreference allele counts and read-depth to account for somatic mutations that were absent in one or both ccfDNA replicates. Overall, 18 of the 29 mutations identified in the tumor tissue had at least one corresponding nonreference allele in pre-operative ccfDNA. However, six of these matches were associated with a nonreference allele count ≤3 (mean VAF of 0.02±0.01%), which likely represented error based on evidence presented above. Thus, these six mutations were scored as absent. Therefore, of the 29 mutations present in tumor, 12 (41.4%) were detected in pre-operative ccfDNA from 8 of the 14 patients (57.1%) with a median VAF of 0.12% (range: 0.04% – 0.33%; Fig. 3B). Data associated with all the tumor mutations at corresponding ccfDNA positions is shown in Table S5. There was no significant difference in pre-operative ccfDNA read-depth between presence/absence of detected mutations (Figure S5). The distribution of VAFs and nonreference allele counts for each variant detected in ccfDNA is shown in Figure S5. In the 11 patients with ccfDNA obtained post-operatively, there were a total of 17 somatic mutations present in tumor tissue DNA. As with the pre-operative ccfDNA, variants with ≤3 nonreference allele counts were considered absent. Six of the 17 (35.3%) somatic mutations were detected in ctDNA from three patients. The identified tumor-guided ctDNA in the post-operative ccfDNA represented a subset of ctDNA detected pre-operatively. Data associated with each tumor mutation at the corresponding position in post-operative ccfDNA are shown in Table S6. Overall, using *a priori* knowledge of patient specific somatic mutations improved detection of ctDNA through relaxation of thresholds intended to maximally suppress noise. However, implementation of an unbiased search enabled discovery of additional somatic mutations not detected in a focal tissue sample of solid tumor.

### Correlation between survival and detection of PDAC-derived ctDNA

**Fig. 4:**
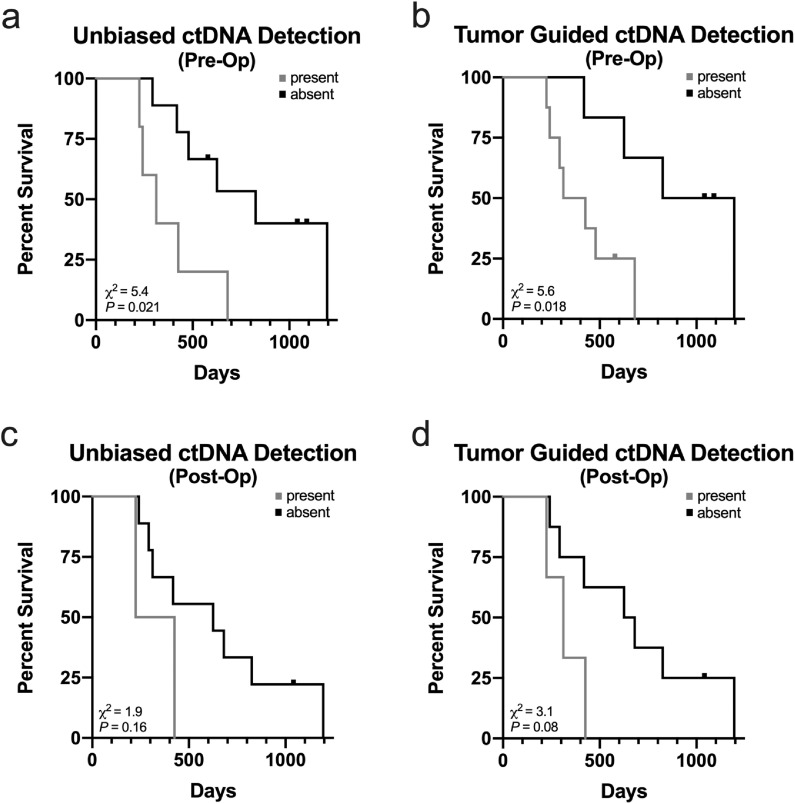
Survival analysis based on presence/absence of ctDNA in ccfDNA acquired pre- and post-operatively in PDAC patients. In (A), technical replicates were integrated into an error suppression strategy to achieve maximum noise reduction across the entire 118 gene panel to identify ctDNA without using somatic mutations identified from corresponding solid tumor DNA to guide detection in pre-operative ccfDNA. In (B), somatic mutations present in tumor DNA were used to guide detection of ctDNA in pre-operative ccfDNA. Detection of ctDNA in pre-operative ccfDNA with and without *a priori* knowledge of somatic mutations yielded similar results. Applying similar strategies for detection of ctDNA in post-operative ccfDNA (C and D, respectively) did not identify a significant difference in survival between patients.

Finally, we evaluated the survival probability based on detection of ctDNA. In the 14 PDAC patients, there was a significant difference between the estimated median survival with (312 days) and without (826 days) detection of ctDNA in pre-operatively obtained ccfDNA using the integrated replicate analysis method for NGS error suppression ($\chi^{2}$=5.4, *P* = 0.021; Fig. 4A). Detection of ctDNA using tumor mutations as a guide also yielded a significant difference between the estimated median survival (369 vs 1,011 days, respectively; $\chi^{2}$=5.6, *P* = 0.018; Fig. 4B). Although there was a trend, a statistically significant difference in survival curves was not observed using post-operatively obtained ccfDNA to detect ctDNA with or without guidance from tumor tissue mutations (Fig. 4C-D, respectively). Thus, using an unbiased search for ctDNA in pre-operatively acquired ccfDNA may provide prognostically important information in a patient with PDAC even before tumor tissue DNA is acquired to enhance the search.

## Discussion

Challenges with distinguishing ctDNA signal from NGS-associated noise have constrained the growth of NGS in ccfDNA diagnostics. In the study described herein, we used NGS to survey for ctDNA across an entire 118 gene panel without *a priori* knowledge of somatic tumor mutations. Successful detection of pathogenic somatic mutations associated with PDAC in ccfDNA was achieved through an integrated noise reduction strategy that used technical replicates to provide maximum error suppression. In so doing, we found PDAC-associated somatic mutations in ccfDNA that were absent in tumor DNA. Moreover, survival curve analysis showed that clinically meaningful information in PDAC patients was present in ccfDNA without requiring solid tumor DNA to guide variant detection. Collectively, these observations provide compelling evidence that combining technical replicates with *in silico* error suppression strategies may enable the broad surveillance for ctDNA and expand the clinical applications of ccfDNA in oncology.

The signal from ctDNA is governed, at least in part, by the ccfDNA input used during library preparation. We targeted 2 mL plasma equivalents of ccfDNA as library input for PDAC patients in this study, which yielded an average pre-operative input of >44 ng of ccfDNA after combining technical replicates. The associated collapsed consensus read-depth was on average >7,300X. Overall, we identified ~41% of the somatic tumor mutations in ccfDNA, which corresponded to detection of ctDNA in ~53% of PDAC patients. We did not observe a difference in library input or read-depth between patients with and without ctDNA detection suggesting that absence of detection was more likely biologic than technical. This latter assertion is supported by evidence from two previous studies that included PDAC and used a multi-gene panel capture-enrichment NGS approach. In 2015, Zill et al. identified ~65% of somatic tumor mutations in PDAC in ccfDNA and found evidence of corresponding ctDNA in >92% of patients with PDACs [8]. In 2017, Pishvaian reported an overall sensitivity for detecting somatic tumor mutations in ccfDNA at 25% and found ~68% of PDAC patients with tumor DNA present in ccfDNA [9]. Because both studies used 1 mL plasma and a ccfDNA library input 5 to 30 ng, the higher per patient detection rate was most likely attributable to the generally more advanced disease studied. In the former study, ~88% of patients had metastatic disease and allele fractions ranged from 0.2% to 38.5%, while the latter study included ~84% of patients with metastatic disease. In our study, <15% of patients had stage IV PDAC, suggesting that the generally higher ccfDNA library input supported detection of somatic tumor mutations in ccfDNA despite the lower disease burden.

Application of alternative NGS-based strategies in earlier stage PDAC have yielded mixed results. Vietsch et al. used an amplicon-based methodology without UMIs that included regions from 56 genes to detect ctDNA associated with stage II PDAC. Out of five patients, 40% had somatic tumor mutations present in *KDR* and/or *KIT* in ccfDNA [10]. Notably, the allele frequency associated with the *KDR* and *KIT* mutations was >40% in both tumor DNA and ccfDNA, which may have been an artifact of selective amplification or identification of single nucleotide polymorphisms because the allele frequency seems disproportionate to disease severity and changed minimally in ccfDNA samples acquired after metastatic disease developed. None of the somatic tumor mutations present in *KRAS, TP53, SMAD4*, or *CDKN2A* were detected in ccfDNA.[10] The absence of ctDNA detection in these common PDAC-associated genes was likely due to a relatively low ccfDNA input – ccfDNA was extracted from only 200 µL of plasma which is one-tenth the volume used in our study for a single technical replicate. Cohen et al. used an amplicon-based NGS strategy with UMIs to assess two *KRAS* codons (G12 and Q61) in ccfDNA from 221 patients with stage I-II PDAC.[5] They found that 30% of patients had somatic tumor mutations detectable in ccfDNA with a median VAF of 0.12% (range: 0.01 to 1.31%) [5], which is similar to our reported findings in a stage II-III PDAC cohort that found ctDNA associated with 4 genes on our 118 gene panel. Liu et al. described a single-stranded DNA library preparation approach to improve detection of ultra-short ccfDNA fragments (<100 bp) during hybrid-capture of targeted regions in 62 genes [11]. In 13 stage II-III PDAC patients, they identified ~30% of somatic tumor mutations (VAF >1%) in ccfDNA and found evidence of corresponding ctDNA in ~69% of patients that were either the *KRAS* p.G12D or p.G12C variants (median allele frequency of 0.12%; range: 0.05 to 0.56%) [11]. Although previous studies have shown a modest size difference in the fragment length between ctDNA and ccfDNA derived from healthy cells [26], [27], [28], the additional steps inherent to single-stranded DNA library preparation to extract the ultra-short ccfDNA fraction may not necessarily improve ctDNA detection.

*KRAS* activating mutations in the G12, G13, and QQ61 codons occur in more than 90% of PDACs. Because these mutations are so prevalent, presence in ctDNA suggests the mutation should concomitantly be identified in solid tumor DNA. In 50 PDACs with matched solid tumor DNA from the primary lesion and ccfDNA, Cohen et al. found that 100% of *KRAS* mutations in the G12 and Q61 codons found in ctDNA were also present in solid tumor DNA [5]. Zill et al., Pishvaian et al., and Liu et al. reported a similarly high concordance between *KRAS* mutations present in ctDNA that also were identified in solid tumor DNA [8,9,11]. In 26 stage II-III PDAC patients, Guo et al. identified a single *KRAS* p.G12D mutation in ctDNA, confirmed by ddPCR, that was absent in solid tumor DNA [12]. In our study, *KRAS* mutations associated with the G12 and Q61 codons in ctDNA were concordant with solid tumor DNA. However, we also identified two patients with the p.G13D mutation in ctDNA that was absent in solid tumor DNA. In previous PDAC studies that included the G13 codon [8], [9], [10], [11], [12], only a single study reported G13 codon mutations in ctDNA [7]. Unlike colon cancer, *KRAS* G13 mutations are rare in PDAC [29]. Thus, the absence of the p.G13D mutation in the focal tissue sample examined suggests the primary lesion may have been heterogenous and/or the ctDNA was detected from tumor progression in a metastasis. We also identified additional ctDNA pathogenic mutations in *TP53* and *ALK* that were not present in solid tumor DNA. Discordance between ctDNA and tumor DNA in genes beyond *KRAS* has been previously described in PDAC, most commonly in *TP53* [9, 11], but also in *SMAD4, CDKN2A*, and *STK11,* among others [11].

Noise is a component of even the highest fidelity NGS platforms and inherent to the library preparation process [30]. Although we found the combination of UMIs, position-specific error modeling, and technical replicates substantially reduced error, additional criteria were necessary to comprehensively eliminate NGS-associated noise during searches for ctDNA without guidance from somatic tumor mutations. Specifically, we constrained results to potential variants that met a nonreference allele count threshold of >3 and corresponded to a pathogenic variant associated with the pancreas within the COSMIC database. Application of these criteria likely reduced sensitivity. Cohen et al. found that 38% of positive samples had only a single mutant template per mL of plasma in their stage I-II PDAC study [5]. After combining technical replicates, our study used 4 mL of plasma and a combined nonreference allele threshold of >7 mutant templates suggesting that some variants were missed. In addition, use of the COSMIC database limited variant detection to only previously reported mutations meaning that private somatic mutations in ccfDNA unique to a patient's cancer were overlooked. However, unique mutations may not have strong therapeutic implications as each is unlikely to have been previously studied. Nevertheless, alterations to future study designs to increase the signal from ctDNA (e.g., ccfDNA from 4 mL of plasma for each technical replicate) relative to the NGS noise would further expand ctDNA detection for diagnosing and monitoring malignancies.

The association between detection of PDAC-derived ctDNA and reduced survival has been well described [11,12,31]. However, previous studies have largely restricted survival analysis to *KRAS* mutations alone in ctDNA [11,12,31]. Notably, Guo et al. reported in 113 PDAC stage II-III patients that survival differences in ctDNA detection were largely driven by detection of the *KRAS* p.G12D mutation in ctDNA rather than other *KRAS* mutations (e.g., p.G12V, p.G12R, p.Q61R, p.G13D, etc.) [12]. Our study found that inclusion of any PDAC-derived pathogenic mutation from the COSMIC database or a mutation that was present concomitantly in matched solid tumor DNA was associated with reduced survival. We also found that using somatic tumor mutations to guide ctDNA detection yielded a higher number of overall ctDNA variants compared to our unbiased approach of integrating technical replicates into *in silico* analysis for error suppression to identify ctDNA. Importantly, however, we did not appreciate a substantial difference in the estimated survival between patients with and without identification of ctDNA between these two strategies. This latter observation suggests that detection of ctDNA in PDAC with or without somatic tumor tissue guidance may yield prognostic information. Finally, we did not find that searching for ctDNA in post-operative ccfDNA improved the survival analysis. However, this is likely because many samples were acquired in the early post-operative period when an abundance of normal ccfDNA was present in relation to the post-surgical inflammatory and wound healing responses which may have suppressed the ctDNA signal. Our data suggest that future studies may benefit from acquiring post-operative samples >30 days after surgery to allow for the quantity of normal ccfDNA to return to pre-operative levels. If accomplished, evaluating post-operative ctDNA to prognosticate survival, assist earlier recurrence detection, guide treatment, and serve as an adjunct to other tumor markers and radiologic studies, may be a practical use of post-operative ctDNA evaluation.

In conclusion, we found that targeting PDAC pathogenic mutations in the COSMIC database in coordination with experimental and *in silico* techniques to suppress NGS-associated error permitted detection of ctDNA without guidance from somatic tumor mutations. Opening the search for ctDNA to the full array of exonic positions on an NGS multi-gene capture-enrichment panel expanded detection of ctDNA beyond limited selections of somatic tumor mutations identified from a finite tissue sample and provided clinically meaningful survival information. Future strategies that further improve the signal-to-noise ratio of ctDNA may enable monitoring of molecular evolution for both common and private mutations, which is a key clinical goal in the drive towards precision medicine.

## Author Contribution

Kajsa E. Affolter: Conceptualization, Methodology, Investigation, Writing-Review & Editing, Funding acquisition. Sabine Hellwig: Conceptualization, Methodology, Investigation, Writing-Review & Editing, Supervision. David A. Nix: Software, Resources, Writing-Review & Editing Mary P. Bronner: Conceptualization, Methodology, Resources, Writing-Review & Editing, Supervision, Project administration, Funding acquisition Alun Thomas: Formal analysis, Writing-Review & Editing. Carrie L. Fuertes: Investigation, Writing-Review & Editing. Cindy L. Hamil: Investigation, Writing-Review & Editing. Ignacio Garrido-Laguna: Conceptualization, Resources, Writing-Review & Editing. Courtney L. Scaife: Resources, Writing-Review & Editing. Sean J. Mulvihill: Resources, Writing-Review & Editing. Hunter R. Underhill: Conceptualization, Methodology, Investigation, Software, Validation, Formal analysis, Resources, Writing-Original Draft, Supervision, Project administration, Funding acquisition

## Appendix Supplementary materials

Image, application 1
