# Supplementary material for: Detection of circulating tumor DNA without a tumor-informed search using next-generation sequencing is a prognostic biomarker in pancreatic ductal adenocarcinoma
Source: Neoplasia. 2021 Jul 21;23(9):859–69. doi: 10.1016/j.neo.2021.06.005 (PMC8322473; doi:10.1016/j.neo.2021.06.005)
Supplement: Supplementary file 1 [file mmc1.docx]

**Table S1. ccfDNA and sequencing metrics – Replicate 1 (Replicate 2)**

| **Participant**  **(Tumor Stage)** | **Control or Pre-Op ccfDNA** | | | | | | **Post-Op ccfDNA** | | | | | | |
| --- | --- | --- | --- | --- | --- | --- | --- | --- | --- | --- | --- | --- | --- |
|  | **mL Plasma** | **ng/mL Plasma** | **Input, ng** | **mL plasma eq.** | **Paired Reads^†^** | **Consensus Read Depth** | **Days*** | **ng/mL Plasma** | **ng/mL Plasma** | **Input, ng** | **mL plasma eq.** | **Paired Reads^†^** | **Consensus Read Depth** |
| Control 1 | 12.5 | 4.3 | 20.0 | 4.7 | 26.2  (28.0) | 2,523  (2,348) | N/A | | | | | | |
| Control 2 | 13.0 | 5.4 | 20.0 | 3.7 | 28.1  (26.6) | 3,072  (2,726) |  |  |  |  |  |  |  |
| Control 3 | 12.0 | 3.8 | 20.0 | 5.3 | 27.7  (33.2) | 3,134  (3,099) |  |  |  |  |  |  |  |
| Control 4 | 13.0 | 5.8 | 20.0 | 3.4 | 27.8  (26.4) | 3,189  (2,702) |  |  |  |  |  |  |  |
| P1 | 9.0 | 30.2 | 60.4 | 2.0 | 58.3  (36.7) | 5,931  (5,299) | 29 | 10.0 | 24.2 | 50.4 | 2.0 | 28.6  (29.9) | 4,401  (4,441) |
| P2 | 7.4 | 9.2 | 20.0 | 2.2 | 54.1  (32.2) | 3,641  (3,236) | 3 | 5.0 | 67.7 | 135.4 | 2.0 | 29.4  (29.7) | 4,649  (4,599) |
| P3 | 8.0 | 15.2 | 30.4 | 2.0 | 59.7  (33.5) | 5,142  (4,480) | 43 | 8.0 | 13.8 | 27.6 | 2.0 | 29.4  (28.1) | 3,912  (3,809) |
| P4 | 9.0 | 5.4 | 20.0 | 3.7 | 45.8  (32.8) | 3,942  (3,840) | 2 | 9.5 | 64.8 | 129.6 | 2.0 | 32.8  (26.3) | 5,134  (1,499) |
| P5 | 8.5 | 15.2 | 30.4 | 2.0 | 50.9  (31.7) | 4,655  (4,190) | 34 | 9.5 | 45.5 | 91.0 | 2.0 | 34.9  (26.9) | 4,910  (1,889) |
| P6 | 7.8 | 8.1 | 20.0 | 2.5 | 50.7  (43.6) | 4,106  (3,923) | 2 | 11.5 | 44.5 | 89.0 | 2.0 | 25.8  (29.6) | 3,934  (3,862) |
| P7 | 8.0 | 3.5 | 8.7 | 2.5 | 27.3  (27.1) | 2,733  (2,642) | 16 | 8.0 | 10.1 | 20.2 | 2.0 | 28.2  (32.3) | 3,552  (3,678) |
| P8 | 9.5 | 7.4 | 20.0 | 2.7 | 26.7  (29.9) | 3,378  (3,567) | 8 | 8.0 | 14.1 | 28.2 | 2.0 | 28.8  (29.2) | 3,699  (3,761) |
| P9 | 4.0 | 4.9 | 9.8  (7.8) | 2.0  (1.6) | 30.1  (28.8) | 2,828  (2,338) | -- | -- | -- | -- | -- | -- | -- |
| P10 | 8.0 | 11.0 | 22.0 | 2.0 | 28.6  (29.1) | 4,059  (1,874) | -- | -- | -- | -- | -- | -- | -- |
| P11 | 6.0 | 6.1 | 15.0 | 2.5 | 27.5  (26.3) | 3,339  (3,052) | -- | -- | -- | -- | -- | -- | -- |
| P12 | 7.5 | 6.9 | 20.0 | 2.9 | 27.9  (29.7) | 3,768  (3,667) | 97 | 9.5 | 19.8 | 39.6 | 2.0 | 26.2  (30.1) | 3,601  (3,997) |
| P13 | 4.2 | 3.5 | 7.0 | 2.0 | 25.2  (25.3) | 2,405  (2,288) | 49 | 8.5 | 3.2 | 12.0 | 3.8 | 26.4  (25.8) | 2,667  (2,769) |
| P14 | 7.5 | 8.4 | 20.0 | 2.4 | 27.2  (31.5) | 3,761  (3,810) | 34 | 9.5 | 13.7 | 27.4 | 2.0 | 52.5  (31.7) | 4,207  (4,173) |

*Number of days after surgery sample was obtained

^†^x1,000,000

-- = sample not obtained

**Table S2. ccfDNA pathogenic variants associated with the pancreas in the COSMIC database from Replicate 1.**

| **Patient** | **Gene** | **cDNA** | **AA** | **NRA Count** | **Read-Depth** | **VAF, %** | **Z-score** |
| --- | --- | --- | --- | --- | --- | --- | --- |
| C1 | *ERBB4* | c.750G>T | p.M250I | 4 | 2710 | 0.15 | 100.0 |
|  | *MET* | c.959C>G | p.A320G | 5 | 3406 | 0.15 | 5.3 |
|  | *TP53* | c.586C>T | p.R196X | 4 | 3336 | 0.12 | 11.4 |
|  | *TP53* | c.524G>A | p.R175H | 6 | 3070 | 0.20 | 5.7 |
|  | *VHL* | c.481C>T | p.R161X | 4 | 2957 | 0.14 | 3.4 |
| C2 | *EGFR* | c.2359C>T | p.R787C | 4 | 3839 | 0.10 | 3.5 |
|  | *MSH6* | c.3163G>A | p.A1055T | 4 | 3835 | 0.10 | 8.8 |
|  | *NF2* | c.433G>A | p.A145T | 5 | 5188 | 0.10 | 2.7 |
| C3 | *NF2* | c.491C>T | p.A164V | 4 | 4373 | 0.09 | 8.3 |
|  | *TP53* | c.818G>A | p.R273H | 4 | 4014 | 0.10 | 100.0 |
|  | *TP53* | c.581T>G | p.L194R | 7 | 4089 | 0.17 | 11.5 |
|  | *TP53* | c.524G>A | p.R175H | 5 | 4322 | 0.12 | 2.9 |
| C4 | *MSH6* | c.1981C>T | p.R661C | 4 | 4566 | 0.09 | 3.2 |
|  | | | | | | | |
| P1 | *KDR* | c.2854G>A | p.V952I | 4 | 8335 | 0.05 | 100.0 |
|  | *KRAS* | c.53C>T | p.A18V | 4 | 7033 | 0.06 | 2.8 |
|  | *TP53* | c.916C>T | p.R306X | 4 | 9858 | 0.04 | 3.8 |
| P2 | *APC* | c.2485A>G | p.T829A | 6 | 4035 | 0.15 | 3.1 |
|  | *APC* | c.3949G>C | p.E1317Q | 4 | 4701 | 0.09 | 3.1 |
|  | *CDKN2A* | c.39G>A | p.L13L | 5 | 1124 | 0.44 | 16.3 |
|  | *GABRB2* | c.919G>A | p.V307I | 4 | 4805 | 0.08 | 3.4 |
|  | *KRAS* | c.181C>A | p.Q61K+ | 17 | 4172 | 0.41 | 10.5 |
|  | *KRAS* | c.38G>A | p.G13D | 119 | 4427 | 2.69 | 100.0 |
|  | *KRAS* | c.35G>A | p.G12D | 4 | 4453 | 0.09 | 4.8 |
|  | *KRAS* | c.34G>C | p.G12R | 5 | 4442 | 0.11 | 100.0 |
|  | *TRAF7* | c.1606G>A | p.G536S | 6 | 3600 | 0.17 | 10.8 |
| P3 | *ERBB4* | c.750G>T | p.M250I | 5 | 6241 | 0.08 | 100.0 |
|  | *KRAS* | c.35G>A | p.G12D | 12 | 6391 | 0.19 | 10.6 |
|  | *PIK3CA* | c.3145G>C | p.G1049R | 4 | 7019 | 0.06 | 100.0 |
|  | *PTEN* | c.202T>C | p.Y68H | 5 | 6010 | 0.08 | 4.7 |
|  | *SMAD4* | c.1082G>A | p.R361H | 16 | 7002 | 0.23 | 6.6 |
|  | *TP53* | c.713G>A | p.C238Y | 4 | 5966 | 0.07 | 4.4 |
|  | *TP53* | c.586C>T | p.R196X | 17 | 5759 | 0.30 | 28.5 |
|  | *TP53* | c.406C>G | p.Q136E | 5 | 5313 | 0.09 | 3.0 |
| P4 | *EGFR* | c.2447T>A | p.L816Q | 4 | 6112 | 0.07 | 4.4 |
|  | *MTOR* | c.10G>A | p.E4K | 4 | 3765 | 0.11 | 100.0 |
|  | *TP53* | c.989T>G | p.L330R | 4 | 6877 | 0.06 | 6.5 |
|  | *TP53* | c.586C>T | p.R196X | 4 | 5420 | 0.07 | 6.9 |
|  | *TRAF7* | c.1606G>A | p.G536S | 6 | 3668 | 0.16 | 10.6 |
| P5 | *CDKN2A* | c.152T>A | p.V51D | 4 | 1832 | 0.22 | 3.0 |
|  | *TP53* | c.641A>G | p.H214R | 4 | 6243 | 0.06 | 100.0 |
| P6 | *ERBB4* | c.317G>A | p.R106H | 4 | 5845 | 0.07 | 100.0 |
|  | *GNAS* | c.680A>T | p.Q227L | 4 | 4664 | 0.09 | 100.0 |
|  | *TP53* | c.751A>T | p.I251F | 4 | 5692 | 0.07 | 3.3 |
|  | *TP53* | c.281C>A | p.S94X | 4 | 3276 | 0.12 | 11.9 |
| P7 | *CDKN2A* | c.140A>C | p.H47P | 4 | 2975 | 0.13 | 100.0 |
|  | *KRAS* | c.38G>A | p.G13D | 26 | 2968 | 0.88 | 100.0 |
|  | *NF1* | c.7873G>A | p.A2625T | 4 | 2877 | 0.14 | 12.2 |
|  | *TP53* | c.637C>T | p.R213X | 5 | 3499 | 0.14 | 3.1 |
| P8 | *PIK3CA* | c.35G>T | p.G12V | 5 | 4140 | 0.12 | 5.2 |
|  | *TP53* | c.535C>T | p.H179Y | 5 | 5015 | 0.10 | 3.1 |
| P9 | *TP53* | c.701A>G | p.Y234C | 4 | 3686 | 0.11 | 5.1 |
|  | *TP53* | c.524G>A | p.R175H | 4 | 3721 | 0.11 | 2.6 |
|  | *TP53* | c.488A>G | p.Y163C | 5 | 3990 | 0.13 | 4.5 |
|  | *VHL* | c.371C>A | p.T124K | 4 | 3641 | 0.11 | 100.0 |
|  | *VHL* | c.481C>T | p.R161X | 4 | 3686 | 0.11 | 2.6 |
| P10 | *APC* | c.4678G>T | p.E1560X | 5 | 5202 | 0.10 | 2.6 |
|  | *TP53* | c.643A>G | p.S215G | 5 | 5598 | 0.09 | 10.0 |
|  | *TP53* | c.430C>T | p.Q144X | 6 | 5057 | 0.12 | 2.9 |
| P11 | *ALK* | c.3300C>A | p.G1100G | 5 | 4947 | 0.10 | 2.8 |
|  | *TP53* | c.818G>A | p.R273H | 4 | 4436 | 0.09 | 100.0 |
|  | *TP53* | c.578A>T | p.H193L | 5 | 4862 | 0.10 | 4.2 |
|  | *TP53* | c.503A>G | p.H168R | 4 | 4789 | 0.08 | 100.0 |
| P12 | *ALK* | c.3257C>T | p.S1086L | 5 | 6104 | 0.08 | 6.8 |
|  | *ATM* | c.3883C>T | p.L1295F | 4 | 5140 | 0.08 | 100.0 |
|  | *FBXW7* | c.1513C>T | p.R505C | 5 | 4987 | 0.10 | 100.0 |
|  | *SMAD4* | c.523G>C | p.E175Q | 4 | 4669 | 0.09 | 2.7 |
|  | *SMAD4* | c.1370G>C | p.X457S | 5 | 3846 | 0.13 | 100.0 |
|  | *TP53* | c.722C>A | p.S241Y | 4 | 6043 | 0.07 | 3.9 |
|  | *TP53* | c.574C>T | p.Q192X | 4 | 5590 | 0.07 | 5.6 |
| P13 | *ALK* | c.3257C>T | p.S1086L | 4 | 3299 | 0.12 | 10.3 |
|  | *NF2* | c.433G>A | p.A145T | 4 | 3734 | 0.11 | 3.1 |
|  | *SMAD4* | c.1216G>A | p.A406T | 4 | 2671 | 0.15 | 4.1 |
|  | *TP53* | c.725G>C | p.C242S | 4 | 3206 | 0.12 | 5.7 |
|  | *TP53* | c.578A>T | p.H193L | 6 | 2697 | 0.22 | 9.7 |
|  | *TP53* | c.452C>A | p.P151H | 7 | 2901 | 0.24 | 4.2 |
| P14 | *KRAS* | c.34G>C | p.G12R | 8 | 4338 | 0.18 | 100.0 |
|  | *NF2* | c.491C>T | p.A164V | 4 | 6052 | 0.07 | 6.0 |
|  | *PTEN* | c.202T>C | p.Y68H | 4 | 4318 | 0.09 | 5.4 |
|  | *SMAD4* | c.394C>T | p.H132Y | 4 | 5371 | 0.07 | 100.0 |
|  | *SPTA1* | c.575G>T | p.R192L | 5 | 4226 | 0.12 | 5.4 |
|  | *TP53* | c.844C>T | p.R282W | 12 | 5575 | 0.22 | 6.0 |
|  | *TP53* | c.455C>G | p.P152R | 4 | 5113 | 0.08 | 5.2 |

AA = amino acid; NRA = nonreference allele; VAF = variant allele frequency

p.Q61K+ (P2) -- the ‘+’ indicates there was a co-occurring c.180T>A mutation

**Table S3. ccfDNA pathogenic variants associated with the pancreas in the COSMIC database from Replicate 2.**

| **Patient** | **Gene** | **cDNA** | **AA** | **NRA Count** | **Read-Depth** | **VAF, %** | **Z-score** |
| --- | --- | --- | --- | --- | --- | --- | --- |
| C1 | *EGFR* | c.2359C>T | p.R787C | 4 | 3899 | 0.10 | 3.5 |
|  | *ERBB4* | c.943G>A | p.V315I | 5 | 3800 | 0.13 | 2.6 |
|  | *SEMA3E* | c.1811C>T | p.T604M | 6 | 4393 | 0.14 | 3.7 |
|  | *TP53* | c.848G>C | p.R283P | 4 | 3929 | 0.10 | 5.9 |
|  | *TP53* | c.430C>T | p.Q144X | 4 | 1896 | 0.21 | 100.0 |
| C2 | *NF2* | c.1021C>T | p.R341X | 5 | 5613 | 0.09 | 5.1 |
|  | *SMAD4* | c.968G>A | p.W323X | 9 | 4425 | 0.20 | 3.1 |
|  | *SPTA1* | c.575G>T | p.R192L | 4 | 3281 | 0.12 | 5.6 |
|  | *TP53* | c.742C>T | p.R248W | 4 | 3198 | 0.13 | 100.0 |
| C3 | *GABRA1* | c.281G>A | p.R94H | 4 | 3969 | 0.10 | 7.5 |
|  | *LZTR1* | c.2471T>A | p.L824Q | 9 | 1553 | 0.58 | 2.6 |
|  | *NF1* | c.1414A>G | p.N472D | 5 | 3167 | 0.16 | 4.9 |
|  | *PIK3CA* | c.1654T>G | p.W552G | 7 | 4291 | 0.16 | 10.8 |
|  | *TP53* | c.848G>C | p.R283P | 4 | 4882 | 0.08 | 4.7 |
|  | *TP53* | c.733G>A | p.G245S | 4 | 4505 | 0.09 | 100.0 |
| C4 | *APC* | c.4348C>T | p.R1450X | 4 | 4872 | 0.08 | 4.5 |
|  | *PIK3CA* | c.3139C>T | p.H1047Y | 4 | 4722 | 0.08 | 100.0 |
|  | *SMAD4* | c.857A>T | p.H286L | 4 | 3816 | 0.10 | 100.0 |
|  | | | | | | | |
| P1 | *ALK* | c.3743G>A | p.R1248Q | 5 | 5211 | 0.10 | 4.6 |
|  | *PIK3CA* | c.35G>T | p.G12V | 6 | 5422 | 0.11 | 4.8 |
|  | *PTEN* | c.697C>T | p.R233X | 4 | 7340 | 0.05 | 100.0 |
|  | *SMAD4* | c.533C>G | p.S178X | 5 | 6447 | 0.08 | 100.0 |
|  | *TP53* | c.736A>G | p.M246V | 6 | 9182 | 0.07 | 100.0 |
| P2 | *KRAS* | c.181C>A | p.Q61K+ | 8 | 3724 | 0.21 | 5.1 |
|  | *KRAS* | c.38G>A | p.G13D | 8 | 3572 | 0.22 | 100.0 |
|  | *SPTA1* | c.6632G>A | p.R2211H | 5 | 4314 | 0.12 | 9.3 |
| P3 | *CTNNB1* | c.113C>T | p.S38F | 4 | 6161 | 0.06 | 100.0 |
|  | *KRAS* | c.35G>A | p.G12D | 17 | 5032 | 0.34 | 19.6 |
|  | *SMAD4* | c.392A>G | p.Y131C | 4 | 5899 | 0.07 | 100.0 |
|  | *SMAD4* | c.1082G>A | p.R361H | 10 | 5301 | 0.19 | 5.4 |
|  | *TP53* | c.701A>G | p.Y234C | 4 | 5277 | 0.08 | 3.2 |
|  | *TP53* | c.586C>T | p.R196X | 24 | 5817 | 0.41 | 40.0 |
| P4 | *CDKN2A* | c.386A>G | p.Y129C | 4 | 4775 | 0.08 | 3.3 |
|  | *CDKN2A* | c.34C>G | p.L12V | 5 | 4466 | 0.11 | 100.0 |
|  | *FBXW7* | c.1394G>A | p.R465H | 4 | 5033 | 0.08 | 2.7 |
|  | *FGFR1* | c.482G>A | p.R161Q | 5 | 5299 | 0.09 | 100.0 |
|  | *TP53* | c.578A>T | p.H193L | 6 | 5614 | 0.11 | 4.4 |
|  | *TP53* | c.482C>A | p.A161D | 4 | 5604 | 0.07 | 3.1 |
| P5 | *ALK* | c.3257C>T | p.S1086L | 4 | 7208 | 0.06 | 4.4 |
| P6 | *NOTCH1* | c.7369C>A | p.L2457M | 5 | 4110 | 0.12 | 100.0 |
|  | *PTEN* | c.466G>T | p.G156W | 4 | 4380 | 0.09 | 100.0 |
|  | *RET* | c.2689C>T | p.R897X | 5 | 5731 | 0.09 | 4.2 |
|  | *SMAD4* | c.403C>T | p.R135X | 4 | 6399 | 0.06 | 100.0 |
|  | *TP53* | c.805A>T | p.S269C | 4 | 5204 | 0.08 | 100.0 |
|  | *TP53* | c.751A>T | p.I251F | 4 | 6415 | 0.06 | 100.0 |
|  | *TP53* | c.527G>A | p.C176Y | 4 | 5440 | 0.07 | 3.0 |
|  | *VHL* | c.481C>T | p.R161X | 9 | 5874 | 0.15 | 3.9 |
| P7 | *ATM* | c.5291T>A | p.L1764Q | 4 | 2465 | 0.16 | 100.0 |
|  | *KRAS* | c.38G>A | p.G13D | 33 | 2730 | 1.21 | 100.0 |
|  | *PIK3CA* | c.1654T>G | p.W552G | 4 | 3162 | 0.13 | 8.3 |
|  | *TP53* | c.821T>A | p.V274D | 4 | 2955 | 0.14 | 11.2 |
|  | *TP53* | c.725G>C | p.C242S | 4 | 3425 | 0.12 | 5.3 |
|  | *TP53* | c.472C>T | p.R158C | 8 | 3508 | 0.23 | 14.9 |
| P8 | *CDKN2A* | c.104C>T | p.A35V | 4 | 5138 | 0.08 | 4.1 |
|  | *RB1* | c.1463C>T | p.A488V | 4 | 3307 | 0.12 | 3.3 |
|  | *SMAD4* | c.1370G>C | p.X457S | 4 | 3709 | 0.11 | 100.0 |
|  | *SPTA1* | c.4241G>T | p.R1414L | 4 | 6011 | 0.07 | 4.7 |
|  | *TP53* | c.542G>A | p.R181H | 4 | 5642 | 0.07 | 2.9 |
| P9 | *NF2* | c.1021C>T | p.R341X | 4 | 3889 | 0.10 | 6.0 |
|  | *TP53* | c.586C>T | p.R196X | 4 | 3414 | 0.12 | 11.1 |
|  | *TP53* | c.523C>G | p.R175G | 4 | 3061 | 0.13 | 4.4 |
| P10 | *ATM* | c.9023G>A | p.R3008H | 7 | 2264 | 0.31 | 8.0 |
|  | *CDKN2A* | c.236T>A | p.L79Q | 4 | 2199 | 0.18 | 4.5 |
|  | *RB1* | c.2224G>A | p.V742I | 4 | 781 | 0.51 | 3.1 |
|  | *SMAD4* | c.1577A>T | p.E526V | 4 | 2538 | 0.16 | 100.0 |
|  | *TP53* | c.1024C>T | p.R342X | 7 | 2201 | 0.32 | 10.5 |
|  | *TP53* | c.481G>A | p.A161T | 5 | 2383 | 0.21 | 7.1 |
|  | *TP53* | c.434T>A | p.L145Q | 4 | 2374 | 0.17 | 100.0 |
| P11 | *ATM* | c.5291T>A | p.L1764Q | 4 | 3541 | 0.11 | 100.0 |
|  | *KRAS* | c.179G>A | p.G60D | 5 | 4062 | 0.12 | 7.2 |
|  | *TP53* | c.589G>A | p.V197M | 5 | 4425 | 0.11 | 9.2 |
| P12 | *ALK* | c.3257C>T | p.S1086L | 4 | 5658 | 0.07 | 5.8 |
|  | *APC* | c.4639G>T | p.E1547X | 4 | 5216 | 0.08 | 100.0 |
|  | *KRAS* | c.35G>A | p.G12D | 4 | 4407 | 0.09 | 4.8 |
|  | *TP53* | c.423C>A | p.C141X | 4 | 5131 | 0.08 | 3.2 |
| P13 | *ATM* | c.8246A>T | p.K2749I | 6 | 2066 | 0.29 | 3.3 |
|  | *SMAD4* | c.379T>A | p.C127S | 4 | 2884 | 0.14 | 100.0 |
|  | *SMAD4* | c.1363C>T | p.Q455X | 5 | 2730 | 0.18 | 4.3 |
|  | *SMAD4* | c.1543A>T | p.R515X | 4 | 2808 | 0.14 | 100.0 |
|  | *TP53* | c.848G>C | p.R283P | 4 | 2642 | 0.15 | 7.8 |
|  | *TP53* | c.578A>T | p.H193L | 4 | 2708 | 0.15 | 9.7 |
|  | *TP53* | c.472C>T | p.R158C | 4 | 2681 | 0.15 | 9.4 |
| P14 | *KRAS* | c.176C>G | p.A59G | 4 | 4794 | 0.08 | 5.2 |
|  | *KRAS* | c.34G>C | p.G12R | 6 | 4408 | 0.14 | 100.0 |
|  | *TP53* | c.844C>T | p.R282W | 7 | 5461 | 0.13 | 3.3 |
|  | *TP53* | c.475G>C | p.A159P | 4 | 5422 | 0.07 | 3.2 |

AA = amino acid; NRA = nonreference allele; VAF = variant allele frequency

p.Q61K+ (P2) -- the ‘+’ indicates there was a co-occurring c.180T>A mutation

**Table S4. Solid tumor DNA somatic mutations in PDAC patients.**

| **Patient** | **Gene** | **cDNA** | **AA** | **NRA Count** | **Read-Depth** | **VAF, %** |
| --- | --- | --- | --- | --- | --- | --- |
| P1 | *KRAS* | c.35G>A | p.G12D | 229 | 4,941 | 4.63 |
| P2 | *KRAS* | c.181C>A | p.Q61K+ | 1945 | 5,154 | 37.74 |
| P3 | *KRAS* | c.35G>A | p.G12D | 552 | 5,092 | 10.84 |
|  | *SMAD4* | c.1082G>A | p.R361H | 304 | 4,848 | 6.27 |
|  | *TP53* | c.586C>T | p.R196X | 771 | 6,474 | 11.91 |
| P4 | *KRAS* | c.35G>A | p.G12D | 706 | 4,313 | 16.37 |
|  | *TP53* | c.752T>C | p.I251T | 1128 | 5,789 | 19.49 |
| P5 | *KRAS* | c.35G>C | p.G12A | 657 | 4,321 | 15.20 |
|  | *KRAS* | c.26T>C | p.V9A | 612 | 4,298 | 14.24 |
|  | *TP53* | c.641A>G | p.H214R | 724 | 4,822 | 15.01 |
| P6 | *KRAS* | c.34G>C | p.G12R | 211 | 2,665 | 7.92 |
| P7 | *KRAS* | c.35G>A | p.G12D | 73 | 2,310 | 3.16 |
| P8 | *KRAS* | c.35G>A | p.G12D | 97 | 1,603 | 6.05 |
| P9 | *CDKN2A* | c.172C>T | p.R58X | 735 | 6,473 | 11.35 |
|  | *KRAS* | c.34G>T | p.G12C | 1331 | 6,431 | 20.70 |
|  | *TP53* | c.524G>A | p.R175H | 826 | 7,556 | 10.93 |
| P10 | *CDKN2A* | c.238C>T | p.R80X | 1468 | 7,557 | 19.43 |
|  | *KRAS* | c.35G>A | p.G12D | 144 | 1,135 | 12.69 |
|  | *SMAD4* | c.343T>A | p.C115S | 16 | 1,354 | 1.18 |
|  | *TP53* | c.743G>A | p.R248Q | 400 | 4,155 | 9.63 |
| P11 | *CDKN2A* | c.238C>T | p.R80X | 349 | 6,987 | 4.99 |
|  | *KRAS* | c.59C>T | p.T20M | 69 | 1,590 | 4.34 |
|  | *KRAS* | c.35G>T | p.G12V | 61 | 1,584 | 3.85 |
|  | *SMAD4* | c.343T>A | p.C115S | 19 | 1,786 | 1.06 |
|  | *TP53* | c.524G>A | p.R175H | 318 | 7,827 | 4.06 |
| P12 | *KRAS* | c.35G>A | p.G12D | 673 | 3,896 | 17.27 |
| P13 | *KRAS* | c.35G>A | p.G12D | 2723 | 4,524 | 60.19 |
| P14 | *KRAS* | c.34G>C | p.G12R | 3297 | 5,903 | 55.85 |
|  | *TP53* | c.596G>T | p.G199V | 2449 | 4,947 | 49.50 |

AA = amino acid; NRA = nonreference allele; VAF = variant allele frequency

p.Q61K+ (P2) -- the ‘+’ indicates there was a co-occurring c.180T>A mutation

**Table S5. Presence/absence of tumor mutations in pre-operative ccfDNA.**

| **Patient** | **Gene** | **cDNA** | | **AA** | | **Tumor VAF, %** | **ccfDNA** | | | | |
| --- | --- | --- | --- | --- | --- | --- | --- | --- | --- | --- | --- |
|  |  |  |  |  |  |  | **NRA Count** | | | **Read-Depth** | **VAF, %** |
| **PRESENT** | | | | | | | | | | | |
| P2 | *KRAS* | c.181C>A | p.Q61K | | 37.7 | | | 25 | 7,789 | | 0.32 |
| P3 | *KRAS* | c.35G>A | p.G12D | | 10.8 | | | 28 | 10,982 | | 0.25 |
|  | *SMAD4* | c.1082G>A | p.R361H | | 6.3 | | | 20 | 11,971 | | 0.17 |
|  | *TP53* | c.586C>T | p.R196X | | 11.9 | | | 37 | 11,247 | | 0.33 |
| P5 | *TP53* | c.641A>G | p.H214R | | 15.0 | | | 4 | 10,479 | | 0.04 |
| P7 | *KRAS* | c.35G>A | p.G12D | | 3.2 | | | 4 | 5,639 | | 0.07 |
| P9 | *CDKN2A* | c.172C>T | p.R58X | | 11.4 | | | 5 | 5,089 | | 0.10 |
|  | *TP53* | c.524G>A | p.R175H | | 10.9 | | | 4 | 6,306 | | 0.06 |
| P11 | *TP53* | c.524G>A | p.R175H | | 4.1 | | | 4 | 6,913 | | 0.06 |
| P12 | *KRAS* | c.35G>A | p.G12D | | 17.3 | | | 6 | 7,476 | | 0.08 |
| P14 | *KRAS* | c.34G>C | p.G12R | | 55.9 | | | 10 | 7,552 | | 0.13 |
|  | *TP53* | c.596G>T | p.G199V | | 49.5 | | | 13 | 9,072 | | 0.14 |
| **ABSENT** | | | | | | | | | | | |
| P1 | *KRAS* | c.35G>A | p.G12D | | 4.6 | | | 1 | 10,720 | | 0.01 |
| P4 | *KRAS* | c.35G>A | p.G12D | | 16.4 | | | 0 | 7,708 | | 0.00 |
|  | *TP53* | c.752T>C | p.I251T | | 19.5 | | | 0 | 10,742 | | 0.00 |
| P5 | *KRAS* | c.35G>C | p.G12A | | 15.2 | | | 2 | 9,045 | | 0.02 |
|  | *KRAS* | c.26T>C | p.V9A | | 14.2 | | | 1 | 8,818 | | 0.01 |
| P6 | *KRAS* | c.34G>C | p.G12R | | 7.9 | | | 1 | 9,083 | | 0.01 |
| P8 | *KRAS* | c.35G>A | p.G12D | | 6.1 | | | 0 | 7,254 | | 0.00 |
| P9 | *KRAS* | c.34G>T | p.G12C | | 20.7 | | | 0 | 5,364 | | 0.00 |
| P10 | *CDKN2A* | c.238C>T | p.R80X | | 19.4 | | | 0 | 6,293 | | 0.00 |
|  | *KRAS* | c.35G>A | p.G12D | | 12.7 | | | 0 | 6,184 | | 0.00 |
|  | *SMAD4* | c.343T>A | p.C115S | | 1.2 | | | 0 | 7,168 | | 0.00 |
|  | *TP53* | c.743G>A | p.R248Q | | 9.6 | | | 0 | 8,592 | | 0.00 |
| P11 | *CDKN2A* | c.238C>T | p.R80X | | 5.0 | | | 0 | 6,286 | | 0.00 |
|  | *KRAS* | c.59C>T | p.T20M | | 4.3 | | | 1 | 7,650 | | 0.01 |
|  | *KRAS* | c.35G>T | p.G12V | | 3.9 | | | 0 | 6,914 | | 0.00 |
|  | *SMAD4* | c.343T>A | p.C115S | | 1.1 | | | 0 | 7,309 | | 0.00 |
| P13 | *KRAS* | c.35G>A | p.G12D | | 60.2 | | | 2 | 4,688 | | 0.04 |

**Table S6. Presence/absence of tumor mutations in post-operative ccfDNA.**

| **Patient** | **Gene** | **cDNA** | **AA** | **Tumor VAF, %** | **ccfDNA** | | |
| --- | --- | --- | --- | --- | --- | --- | --- |
|  |  |  |  |  | **NRA Count** | **Read-Depth** | **VAF, %** |
| **PRESENT** | | | | | | | |
| P3 | *KRAS* | c.35G>A | p.G12D | 10.8 | 29 | 8734 | 0.33 |
| P3 | *TP53* | c.586C>T | p.R196X | 11.9 | 27 | 9371 | 0.29 |
| P3 | *SMAD4* | c.1082G>A | p.R361H | 6.3 | 15 | 9736 | 0.15 |
| P12 | *KRAS* | c.35G>A | p.G12D | 17.3 | 8 | 7897 | 0.10 |
| P14 | *KRAS* | c.34G>C | p.G12R | 55.9 | 9 | 8550 | 0.11 |
| P14 | *TP53* | c.596G>T | p.G199V | 49.5 | 6 | 9593 | 0.06 |
| **ABSENT** | | | | | | | |
| P1 | *KRAS* | c.35G>A | p.G12D | 4.6 | 2 | 8042 | 0.02 |
| P2 | *KRAS* | c.181C>A | p.Q61K+ | 37.7 | 2 | 11133 | 0.02 |
| P4 | *KRAS* | c.35G>A | p.G12D | 16.4 | 0 | 6886 | 0.00 |
| P4 | *TP53* | c.752T>C | p.I251T | 19.5 | 1 | 8904 | 0.01 |
| P5 | *KRAS* | c.35G>C | p.G12A | 15.2 | 0 | 6989 | 0.00 |
| P5 | *KRAS* | c.26T>C | p.V9A | 14.2 | 1 | 6859 | 0.01 |
| P5 | *TP53* | c.641A>G | p.H214R | 15.0 | 1 | 8323 | 0.01 |
| P6 | *KRAS* | c.34G>C | p.G12R | 7.9 | 0 | 9258 | 0.00 |
| P7 | *KRAS* | c.35G>A | p.G12D | 3.2 | 0 | 7295 | 0.00 |
| P8 | *KRAS* | c.35G>A | p.G12D | 6.1 | 3 | 7861 | 0.04 |
| P13 | *KRAS* | c.35G>A | p.G12D | 60.2 | 1 | 5907 | 0.02 |
